# Optimizing event-driven spiking neural network with regularization and cutoff

**DOI:** 10.3389/fnins.2025.1522788

**Published:** 2025-02-19

**Authors:** Dengyu Wu, Gaojie Jin, Han Yu, Xinping Yi, Xiaowei Huang

**Affiliations:** ^1^Department of Computer Science, University of Liverpool, Liverpool, United Kingdom; ^2^State Key Laboratory of Computer Science, Institute of Software, CAS, Beijing, China; ^3^Department of Electrical Engineering, Chalmers University of Technology, Gothenburg, Sweden; ^4^National Mobile Communications Research Laboratory, Southeast University, Nanjing, China

**Keywords:** spiking neural network, ANN-to-SNN conversion, SNN regularization, SNN cutoff, adaptive inference

## Abstract

Spiking neural networks (SNNs), which are the next generation of artificial neural networks (ANNs), offer a closer mimicry to natural neural networks and hold promise for significant improvements in computational efficiency. However, the current SNN model is trained to infer over a fixed duration, thereby overlooking the potential for dynamic inference in the SNN model. In this paper, we strengthen the relationship between SNN and event-driven processing by proposing the inclusion of a cutoff in SNN, that can terminate SNN at any time during inference to achieve efficient inference. Two novel optimization techniques are presented to achieve an inference-efficient SNN: a Top-K cutoff and regularization. The proposed regularization influences the training process by optimizing the SNN for the cutoff, whereas the Top-K cutoff technique optimizes the inference phase. We conducted an extensive set of experiments on multiple benchmark frame-based datasets, such as CIFAR10/100, Tiny-ImageNet, and event-based datasets, including CIFAR10-DVS, N-Caltech101, and DVS128 Gesture. The experimental results demonstrate the effectiveness of the proposed techniques in both the ANN-to-SNN conversion and direct training, enabling SNNs to require 1.76 to 2.76 × fewer timesteps for CIFAR-10, while achieving 1.64 to 1.95× fewer timesteps across all event-based datasets, with near-zero accuracy loss. These findings affirm the compatibility and potential benefits of the proposed techniques in terms of enhancing accuracy and reducing inference latency when integrated with existing methods. Code available: https://github.com/Dengyu-Wu/SNNCutoff.

## 1 Introduction

Spiking neural networks (SNNs) have recently attracted significant research and industrial interest because of their energy efficiency and low latency, and there are neuromorphic chips, such as Loihi (Davies et al., 2018) and TrueNorth (Akopyan et al., 2015) on which SNN can be deployed. Mechanistically, the SNN mimics biological neurons that independently process and forward spikes. With this asynchronous working mechanism, only a small subset of neurons is activated during inference. In essence, an SNN is inherently efficient in terms of computation.

The asynchronous mechanism indicates that the proposed SNN is more effective when used with event-based inputs. Neuromorphic sensors, such as the dynamic vision sensor (DVS) proposed by Lichtsteiner et al. (2008), Delbrück et al. (2010), and Gallego et al. (2020), and the dynamic audio sensor (DAS) proposed by Anumula et al. (2018), were developed to generate binary “events,” which are ideal inputs to SNNs. For instance, unlike conventional frame-based cameras, which measure the “absolute” brightness at a constant rate, DVS cameras are bio-inspired sensors that *asynchronously* measure per-pixel brightness changes and output a stream of events that encode the time, location, and sign of the brightness changes (Gallego et al., 2020). DVS adapts dynamically to the scene's activity, with fewer events in static scenes and higher volumes when significant changes occur. Consequently, the energy and bandwidth consumption scale efficiently based on the actual demand (Amir et al., 2017; Kim et al., 2021), and leads to and SNN operates in a sparse manner (Messikommer et al., 2020). However, an additional encoding step, such as rate-based coding (Rueckauer et al., 2017) and Poisson's code (Sengupta et al., 2019), is necessary for frame-based input before forward propagation in the SNN. Irrespective of the input type—event-based or frame-based—SNN can deliver sequential predictions at their outputs, demonstrating that they can predict at any timestep. To exploit these features, we explored the cutoff optimal SNN, which allowed termination at any time during inference on a spike train (i.e., input) and returned the best possible inference result.

One approach to train as SNN is through ANN-to-SNN conversion, which leverages the mature training regime of the ANN to first train a high-accuracy ANN and then convert it into an SNN. The proposed method has led to research focused on achieving near-zero conversion loss (Deng and Gu, 2021; Bu et al., 2022; Han et al., 2020). Another methodology involves the use of backpropagation in SNN training (Wu et al., 2018, 2019; Fang et al., 2021a,b). Due to the non-differentiable nature of spiking, this approach requires the deployment of a surrogate gradient (Wu et al., 2018, 2019). In this paper, we explore a novel SNN cutoff mechanism and propose general optimization strategies for this process. Our goal is to develop an optimal SNN that effectively balances accuracy and latency.

**Figure 1:**
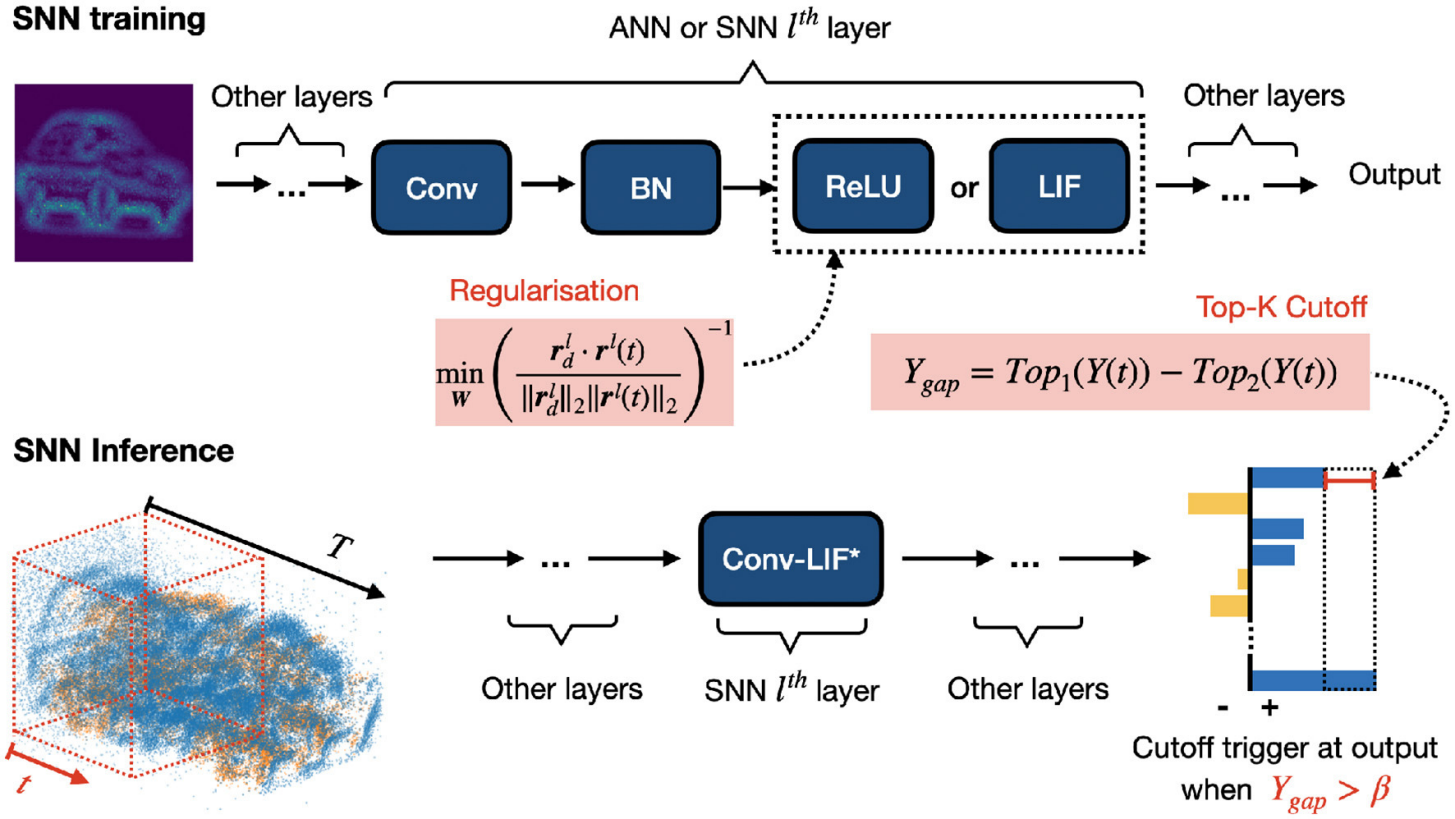
Illustrative diagram showing the regularization process for optimizing the SNN and the cutoff mechanism for reducing latency on the CIFAR10-DVS dataset. The cutoff value is triggered when *Y*_gap_ is greater than β, a value dynamically determined by at a confidence rate introduced in Section 4.1.

This paper makes two key technical contributions. First, instead of always predicting at the maximum timestep *T*, we explore an early cutoff mechanism that allows the SNN model to achieve an optimal latency and computing efficiency automatically. As shown in Figure 1, the SNN model runs a monitoring mechanism to determine whether the decision-makers are sufficiently confident. Once this decision is made at timestep *t*<*T*, i.e., *t*∈{1, 2, …, *T*}, a cutoff action is triggered so that the SNN does not take future inputs until *T*. Therefore, the proposed approach has lower latency and fewer computations because a decision is made at time *t* rather than time *T*.

The second contribution is the proposed regularization technique, which improves the SNN cut-off performance. This technique influences the activation distribution during ANN or SNN training, resulting in an SNN that can classify with less input information. As discussed in Method (Section 4), the proposed regularizer effectively mitigates the impact of “worst-case” inputs during both the ANN and SNN training phases. These worst-case samples are typically inputs that can cause failures in early inference. The experiments presented in Section 5.2 demonstrate that state-of-the-art methods can be enhanced, including ANN-to-SNN conversion and direct training.

To facilitate the analysis, we use the following notations **bold symbol** represents a vector, *l* denotes the layer index, and *i* denotes the element index. For example, ***a***^*l*^ is a vector, and $a_{i}^{l}$ is the *i*-th element in ***a***^*l*^. ***W***^*l*^ is weight matrix at the *l*-th layer.

## 2 Related work

The implementation of SNN involves two phases: training and inference. The training algorithms for SNN can be broadly categorized into two main approaches: ANN-to-SNN conversion and direct training.

### 2.1 ANN-to-SNN conversion

The ANN-to-SNN conversion is a widely studied approach for converting a pre-trained ANN into an SNN model. This process relies on the average spiking rate of neurons, which is closely linked to the normalized activation of the rectified linear unit (ReLU) function in the ANN (Rueckauer et al., 2017). Early studies on ANN-to-SNN conversion, such as Diehl et al. (2015) and Rueckauer et al. (2017), utilized the maximum activation value in each layer of the ANN to normalize the corresponding weights. Sengupta et al. (2019) demonstrated an alternative approach, where normalization can be achieved by greedily searching for the optimal threshold using an input spike train. A unified conversion framework was proposed by Wu et al. (2022), which incorporates a scaling factor that can be applied to either the threshold or weights. Additionally, this framework includes thresholding for residual elimination to mitigate information loss at the last time step, which further enhances conversion efficiency. In a recent study, Deng and Gu (2021) and Wu et al. (2022) demonstrated that outlier elimination in ANN activations can be implemented by applying a clipping operation after ReLU. Based on this, Li et al. (2021a) and Bu et al. (2022) further minimized the quantization error by employing quantization-aware training. In addition, there are other hybrid methods were used to fine-tune the weights in the converted SNN. For example, Rathi et al. (2020) and Rathi and Roy (2021) combined conversion and direct training. Tandem Learning (Wu et al., 2021) leveraged the gradient from the ANN to update the SNN during training.

### 2.2 Direct training

In contrast to conversion-based approaches, direct training of an SNN allows the processing of temporal features effectively (Fang et al., 2021b; Yao et al., 2021). Numerous studies have focused on designing surrogate gradients (Wu et al., 2018, 2019; Neftci et al., 2019; Li et al., 2021b) to tackle the non-differentiable nature of spike generation in SNN, thereby enabling efficient backpropagation. Yao et al. (2021) demonstrated how integrating temporal attention can significantly bolster SNN performance. Advancements in temporal batch normalization (Kim and Panda, 2021; Duan et al., 2022) have been proposed to normalize the input current to SNN layers, thereby expediting the convergence process during direct training. Meanwhile, Guo et al. (2023b) introduced an additional membrane potential normalization, applied after updating the membrane potential with the input current.

Alongside direct training, additional training optimizations, such as knowledge distillation from ANNs (Guo et al., 2023a; Zhang et al., 2024), have been explored as complementary methods to enhance SNN representations. Furthermore, spike-based transformers (Zhou et al., 2023; Zhang et al., 2022; Wang et al., 2023) provide new perspectives by adapting the self-attention mechanism for spike-based computation, replacing complex multiplications with efficient spike-based operations using spike-form queries, keys, and values. Transitioning from conventional binary spikes to ternary spikes (including negative spikes) has also demonstrated performance gains with negligible increases in energy cost, as reported by Guo et al. (2024). Additionally, shrinking the maximum inference timestep as the layers deepen effectively reduces the average inference latency (Ding et al., 2024).

### 2.3 Adaptive inference in SNN

Despite these significant advancements in training algorithms, a fundamental limitation persists: the optimization of SNN is predominantly focused on specific inference durations, neglecting the potential for adaptive inference. The exploration of adaptive inference in SNN is still in its early stages, and only a limited number of studies. Specifically, Li et al. (2023b) introduced an additional deep network to trigger early exiting in an SNN, which is a solution that may be resource-intensive for small SNNs. Similarly, Li et al. (2023a) focused on ANN-to-SNN conversion by applying network calibration before dynamic prediction, while Chen et al. (2023) employed conformal prediction (Shafer and Vovk, 2008) as a trigger mechanism. However, these approaches did not address the optimization of SNN training for such scenarios requiring dynamic timesteps. Our study aimed to fill this gap by combining the cutoff with a general regularizer.

## 3 Leaky integrate-and-fire model

**Figure 2:**
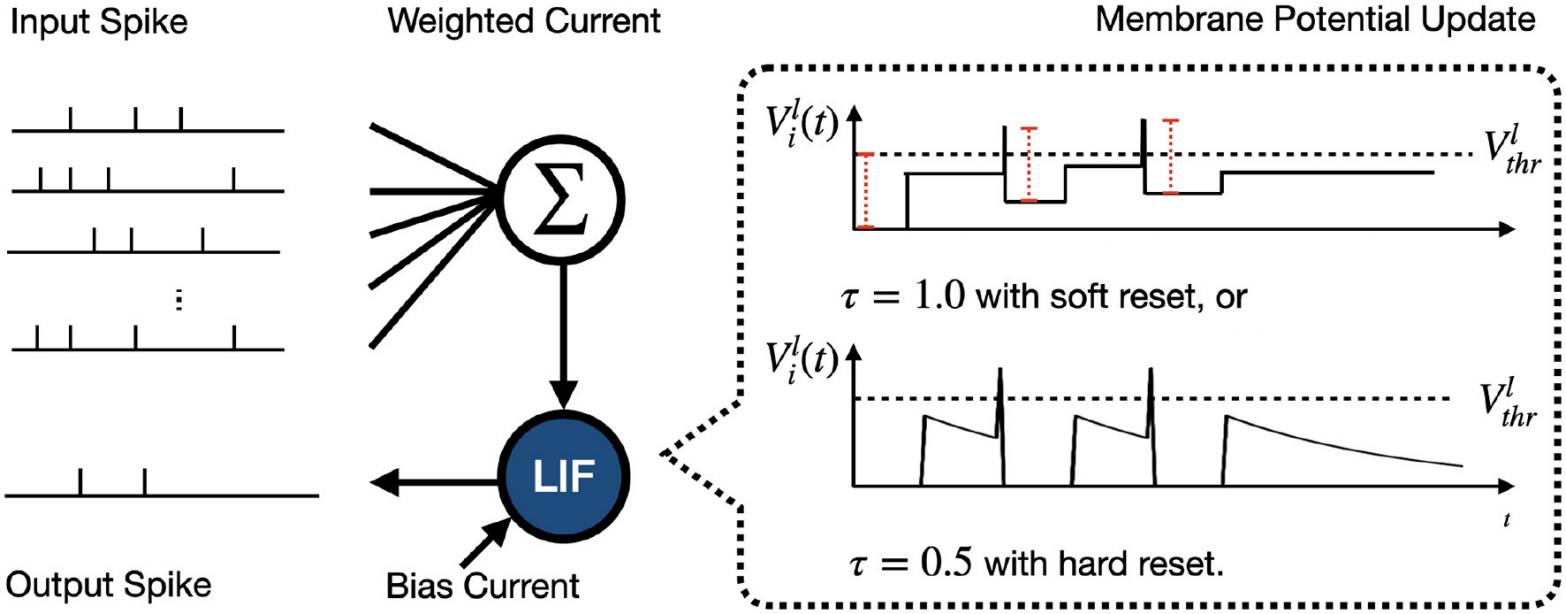
Illustration of inference process for an LIF neuron within the hidden layer. The input spikes charge the membrane potential $V_{i}^{l}\left(t\right)$ through weighted and bias currents learned during training. When $V_{i}^{l}\left(t\right)$ reaches the threshold $V_{\text{thr}}^{l}$, the neuron generates a spike and then resets $V_{i}^{l}\left(t\right)$. In our study, the Integrate-and-Fire (IF) neuron with soft reset is a special case of the LIF model when τ = 1.

The Leaky Integrate-and-Fire (LIF) model is a foundational component in SNN studies and is lauded for its simplicity and resemblance to biological neural processing. Figure 2 illustrates the inference process for a single LIF neuron. The dynamic updates in the LIF neuron are described as


(1) $$V^{l-}(t)=\{\begin{array}{ll} V^{l+}(t-1)+Z^{l}(t), & \text{for }l>1,\\ Z^{1}(t), & \text{for }l=1, \end{array}$$

where ***V***^*l*−^ and ***V***^*l*+^ denote the vector of membrane potentials before and after reset, respectively. The process of resetting of $V_{i}^{l}\left(t\right)$ is categorized as


(2) $$V_{i}^{l+}(t)=\{\begin{array}{ll} \tau V_{i}^{l-}(t)(1-\theta_{i}^{l}(t)), & \text{for hard reset,}\\ \tau V_{i}^{l-}(t)-V_{\text{thr}}^{l}\theta_{i}^{l}(t), & \text{for soft reset,} \end{array}$$

where ***θ***^*l*^(*t*) is a step function, i.e., $\theta_{i}^{l}\left(t\right)=1$ if $V_{i}^{l}\left(t\right)\geq V_{\text{thr}}^{l}$ and $\theta_{i}^{l}\left(t\right)=0$ otherwise. τ is decay factor and soft reset is introduced for ANN-to-SNN conversion. Specifically, conversion-based SNN inference with Integrate-and-Fire (IF) neuron (τ = 1.0) with soft reset aimed at reducing the information loss caused by the conversion (Rückauer et al., 2019; Han et al., 2020; Wu et al., 2022).

In the hidden layers, the weighted current ***Z*****^*l*^**(*t*) is given by


(3) $$\begin{array}{l} Z^{l}\left(t\right)=W^{l}\theta^{l-1}\left(t\right)+b^{l}\text{ when }l>1, \end{array}$$

where ***W***^*l*^ is the weight matrix, and ***b***^*l*^ is the bias current. According to different inputs, ***Z***^*l*^(*t*) at the first layer, i.e., ***Z***^1^(*t*), can be initialized as either


(4) $$\begin{array}{l} Event-based\;input:\;Z_{e}^{1}\left(t\right)=W^{1}X\left(t\right)+b^{1}, \end{array}$$

where ***X***(*t*) is the time-dependent spike train, i.e., the input may change the charging current with time during the inference, or


(5) $$\begin{array}{l} Frame-based\;input:\;Z_{f}^{1}=W^{1}\bar{X}+b^{1}, \end{array}$$

where $\bar{X}$ represents the constant current based on inputs, e.g., normalized pixel value of RGB Image. For each timestep, the first layer of SNN transforms the frame-based input $\bar{X}$ into weighted current $Z_{f}^{1}$, which then stimulates the LIF neurons in the first layer to generate spikes. Notably, for event-based input, SNN can manifest faster inference due to immediate response after receiving the first spike, and it completes the inference whenever the spike train ends, i.e., at *T*. The event-based benchmarks are introduced in Section 5.1. This characteristic allows the inference time to be dynamic for different inputs. In this paper, with the cutoff technique as in Section 4.1, we will show that the average latency of the inference in SNN can be further reduced (to some *t* ≤ *T*).

## 4 Methods

Section 4.1 presents the theoretical underpinnings of cutoff mechanism for the inference. Subsequently, Section 4.2 details the design of a general regularizer to optimize SNN regarding the cutoff mechanism.

### 4.1 Cutoff mechanism in SNN

Owing to its asynchronous working mechanism, the event-driven SNN can predict when only part of the spike train is processed. However, a naive cutoff of the spike train length (or the event sensor's sampling time) can easily lead to a loss of accuracy. In this section, we propose a principled method to determine inference time.

Our approach begins with a theoretical analysis of the cutoff in Section 4.1.1, where we identify the optimal cutoff timestep for each input. Specifically, we explore the optimal timesteps that consistently allow the SNN to make correct predictions in subsequent processing. This evaluation can only be performed in simulations and requires knowledge of future predictions.

To approximate this process in practice, we introduce the Top-K cutoff in Section 4.1.2. To elaborate, we define a confidence rate, denoted as *C*(*t, D*{*Y*_gap_>β_*t*_}), based on the statistical characteristics of processing a set *D* of inputs with respect to the discrete time *t* and the gap between the logits of output neurons, *Y*_gap_. The condition *Y*_gap_>β_*t*_ is used to identify the samples in *D* that are suitable for cutoff. We can plot a curve of the confidence rate *C*(*t, D*{*Y*_gap_>β_*t*_}) with respect to time *t* and constant values β_*t*_. A set of β_*t*_ is extracted from the training samples to trigger the Top-K cutoff.

#### 4.1.1 Optimal cutoff timestep

To define a theoretically optimal cutoff within SNN, we propose identifying the cutoff point where subsequent predictions remain positively true. This criterion establishes the cutoff as the minimal necessary duration of input processing required to uphold predictive reliability. Thus, we define optimal cutoff timestep (OCT) as the smallest timestep $\hat{t}\in \left\{1,2...,T\right\}$ at which the SNN prediction function *f*(***X***, *t*) remains correct prediction for all future timesteps *t* greater than $\hat{t}$, formulated as


(6) $$\begin{array}{l} \text{OCT}\left(X\right)=\min\left\{\hat{t}|\forall t>\hat{t},f\left(X,t\right)\text{is correct}\right\}. \end{array}$$

This equation expresses the earliest timestep from which the prediction function *f*(·) can be accurately and correctly classified according to the partial input. The function OCT(***X***) represents a theoretical lower bound of the average inference timestep, ensuring that each input sample can undergo a minimal inference timestep without sacrificing accuracy.

In our evaluations, we used the OCT as a new metric to assess the performance of SNN models in terms of their cutoff efficiency. This metric allows us to quantitatively determine the lower boundary of the cutoff for any given sample, thereby pinpointing the earliest timestep at which our SNN models can deliver reliable predictions.

#### 4.1.2 Top-K gap for cutoff approximation

During runtime inference, a critical challenge emerges due to the unpredictability of future predictions, which makes determining the OCT in real time impractical. To address this issue, we introduce a cutoff mechanism based on the gap between the largest (top-1) and second-largest (top-2) output logits. This “Top-K” approach suggests that a larger gap between these two output logits indicates a low likelihood of changing the prediction during inference, thereby marking an appropriate point for cutoff.

To formalize this concept, let Top_*k*_(***Y***(*t*)) be the top-*k* logit of one neuron at the output layer. We define the function *Y*_gap_ to represent the gap of top-1 and top-2 values of output ***Y***(*t*) as


(7) $$\begin{array}{l} Y_{\text{gap}}={\text{Top}}_{1}\left(Y\left(t\right)\right)-{\text{Top}}_{2}\left(Y\left(t\right)\right). \end{array}$$

Then, we let *D*{·} denote the inputs in subset of *D* that satisfy a certain condition. Now, we can define the confidence rate *C*(*t, D*{*Y*_gap_>β_*t*_}) as


(8) $$\begin{array}{l} Confidence\;rate:C\left(t,D\left\{Y_{\text{gap}}>\beta_{t}\right\}\right)=\\ \frac{1}{\left|D\left\{Y_{\text{gap}}>\beta_{t}\right\}\right|}\underset{X\in D\left\{Y_{\text{gap}}>\beta_{t}\right\}}{\sum }\left(\text{OCT}\left(X\right)\leq t\right). \end{array}$$

The confidence rate intuitively computes the percentage of inputs in *D* that can achieve the prediction success at or before *T*. |*D*{*Y*_gap_>β_*t*_}| denotes the number of samples in *D* satisfying the condition. It is not hard to see that when *t* = 0, *C*(*t, D*{*Y*_gap_>β_*t*_}) is also 0, and with the increase in time *t*, *C*(*t, D*{*Y*_gap_>β_*t*_}) will also increase until reaching 1. Our algorithm searches for a minimum $\beta_{t}\in {\mathbb{R}}^{+}$ at a specific *t*, as expressed in the following optimization objective


(9) $$\begin{array}{l} \arg\underset{\beta_{t}}{\min}C\left(t,D\left\{Y_{\text{gap}}>\beta_{t}\right\}\right)\geq 1-\epsilon , \end{array}$$

where ϵ is a pre-specified constant such that 1−ϵ represents an acceptable level of confidence for activating cutoff.

**Figure 3:**
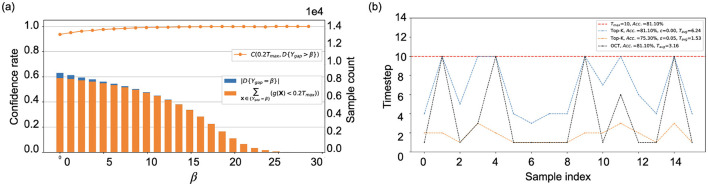
Evaluation of the Top-K cutoff on CIFAR10-DVS, using a directly trained SNN model with *T* = 10 (as detailed in Section 5): **(A)** The increase of β limits the number of samples eligible for cutoff, while concurrently enhancing the confidence in the cutoff decision. **(B)** To enhance the readability, the inference timestep of 16 samples from the test dataset under varied trigger conditions.

Figure 3 provides a visual representation of Equations 6–9, illustrating the theoretical concepts comprehensively. Additionally, Figure 3B highlights the performance disparity between OCT the and Top-K cutoff. The OCT identifies the minimum theoretical timestep $\hat{t}$ for each sample, ensuring that predictions remain accurate as if processed until the maximum timestep *T*. Adjusting the ϵ parameter in the Top-K cutoff allows for a reduced average timestep; however, this may lead to a compromise in accuracy. This insight directs our primary optimization goal toward enhancing SNN cutoff performance, a significant departure from traditional SNN optimizations (Bu et al., 2022; Deng et al., 2022; Yao et al., 2021; Fang et al., 2021a,b) that typically emphasize inference over a fixed duration.

### 4.2 Optimizing SNN for cutoff

To improve the cutoff performance, we concentrated on maximizing the cosine similarity between the actual spiking rate at time *t*, ***r***^*l*^(*t*), and the desired spiking rate, ${\tilde{r}}^{l}$. This objective is achieved by minimizing the inverse of the cosine similarity between these two rates across hidden layers during training, formalized through the introduction of the regularizer of cosine similarity (RCS), defined as


(10) $$\begin{array}{l} \underset{W}{\min}{\left(\frac{{\tilde{r}}^{l}\cdot r^{l}\left(t\right)}{∥{\tilde{r}}^{l}{∥}_{2}∥r^{l}\left(t\right){∥}_{2}}\right)}^{-1}, \end{array}$$

where ${\tilde{r}}^{l}$ is desired spiking rate, and ***r***^*l*^(*t*) denotes the spiking rate at time *t*. Both spiking rates were approximated differently according to conversion (Section 4.2.1) and direct training (Section 4.2.2).

The motivation for using cosine similarity lies in its proven correlation with the final accuracy of quantised neural networks, as demonstrated by Banner et al. (2018). In a similar vein, we hypothesized that a higher cosine similarity between ${\tilde{r}}^{l}$ and ***r***^*l*^(*t*) would correlate with a smaller accuracy drop at time *t*. However, approximating these spike rates poses a significant challenge, particularly within the frameworks of ANN-to-SNN conversion and direct training.

To address these challenges, we differentiate the spiking rates in the context of conversion and direct training as follows ${\tilde{r}}_{c}^{l}$ and $r_{c}^{l}\left(t\right)$ denote the desired and actual spiking rates at time *t*, respectively, in the conversion-based method with IF neurons, while ${\tilde{r}}_{d}^{l}$ and $r_{d}^{l}\left(t\right)$ are used for direct training with LIF neurons. Each method necessitates a distinct approach to approximate these rates, reflecting their unique operational contexts.

It is important to note that there is no established evidence that Equation 10 directly optimizes accuracy at maximum timestep *T*. This is because the cosine similarity term primarily serves as a penalty for alignment between features at different timesteps rather than an explicit measure of distance to the ground truth. Therefore, to avoid any potential degradation in the original training performance, our RCS is applied selectively, focusing only on those samples that yield correct predictions at *T*.

#### 4.2.1 Regularizing ANN before conversion

For conversion-based SNN, Wu et al. (2022) introduced on a fundamental relationship between spiking rates $r_{c}^{l}\left(t\right)$ and ReLU activation ***a***^*l*^, which gives


(11) $$r_{c}^{l}(t)=\frac{1}{V_{\text{thr}}^{l}}(W^{l}r_{c}^{l-1}(t)+b^{l})-\Delta^{l}(t),$$

where $r_{c}^{l}\left(t\right)=\frac{1}{t}\overset{t}{\sum }\theta^{l}\left(t^{\prime }\right)$ denotes the spiking rate at time *t*, with *t*′ representing each discrete timestep leading up to *t* at the at *l*-th layer, and $\Delta^{l}\left(t\right)≜V^{l+}\left(t\right)/\left(tV_{\text{thr}}^{l}\right)$ represents the residual spiking rate.

The spiking rate in the first layer can be initialized as $r_{c}^{1}\left(t\right)=a^{1}/V_{thr}^{1}-\Delta^{1}\left(t\right)$. When *t* is sufficiently large to make **Δ**^*l*^(*t*) negligible, we have the desired spiking rate for the *l*-th layer as


(12) $$\begin{array}{l} {\tilde{r}}_{c}^{l}=\frac{a^{l}}{V_{thr}^{1}}. \end{array}$$

Given that $r_{c}^{l}\left(t\right)={\tilde{r}}_{c}^{l}-\Delta^{l}\left(t\right)$, the cosine similarity is given by


(13) $$\begin{array}{r} \frac{{\tilde{r}}_{c}^{l}\cdot r_{c}^{l}\left(t\right)}{∥{\tilde{r}}_{c}^{l}{∥}_{2}∥r_{c}^{l}\left(t\right){∥}_{2}}\geq \frac{∥{\tilde{r}}_{c}^{l}{∥}_{2}}{∥{\tilde{r}}_{c}^{l}{∥}_{2}+∥\Delta^{l}\left(t\right){∥}_{2}}\\ =\frac{∥a^{l}{∥}_{2}}{∥a^{l}{∥}_{2}+∥V^{l+}\left(t\right)/\left(t\right){∥}_{2}}. \end{array}$$

Assuming that elements in ***V***^*l*+^(*t*) satisfy uniform distribution over the time *t* and they are in [0, *V*_thr_], we can derive boundary for expected value of $∥V^{l+}\left(t\right)/t{∥}_{2}$ as $E\left(∥V^{l+}\left(t\right)/t{∥}_{2}\right)\leq \sqrt{n^{l}}V_{\text{thr}}/\left(\sqrt{3}t\right)$ (proof in Supplementary Material). Moreover, at high dimensions, the relative error made as considering $E\left(∥V^{l+}\left(t\right)/t{∥}_{2}\right)$ instead of the random variable $∥V^{l+}\left(t\right)/t{∥}_{2}$ becomes asymptotically negligible (Biau and Mason, 2015). Therefore, the lower bound of Equation 13 is given by


(14) $$\begin{array}{r} \frac{{\tilde{r}}_{c}^{l}\cdot r_{c}^{l}\left(t\right)}{∥{\tilde{r}}_{c}^{l}{∥}_{2}∥r_{c}^{l}\left(t\right){∥}_{2}}\geq \frac{∥a^{l}{∥}_{2}}{∥a^{l}{∥}_{2}+\sqrt{n^{l}}V_{\text{thr}}^{l}/\left(\sqrt{3}t\right)}\\ =\frac{\sqrt{3}t}{\sqrt{3}t+\sqrt{n^{l}}V_{\text{thr}}^{l}/∥a^{l}{∥}_{2}}. \end{array}$$

This equation explicitly explains that: (a) the increase of *t* to $t\gg \sqrt{n^{l}}V_{\text{thr}}^{l}/∥a^{l}{∥}_{2}$, where *n*^*l*^ is the number of elements in activation ***a***^*l*^, can increase the lower bound, and (b) it is possible to minimize term $\sqrt{n^{l}}V_{\text{thr}}^{l}/∥a^{l}{∥}_{2}$ for developing an SNN with optimized performance at any time during the inference. In other words, for a conversion-based SNN to achieve optimal cutoff performance, the model expects a good (small) ratio of threshold voltage $V_{\text{thr}}^{l}$ to average accumulated current, i.e., $∥a^{l}{∥}_{2}/\sqrt{n^{l}}$, while not degrading SNN classification performance.

In the ANN training, we aim to affect the training process to result in SNN for optimal cutoff. For this purpose, Algorithm 1 was designed to increase the lower bound defined in Equation 14. Following Rueckauer et al. (2017) and Wu et al. (2022), we use the maximum activation value, denoted as *A*_*max*_ in the algorithm to approximate the threshold voltage $V_{\text{thr}}^{l}$ at each layer. The algorithm optimizes activation ratios within each layer during training, directly enhancing SNN performance.

**Algorithm 1:**
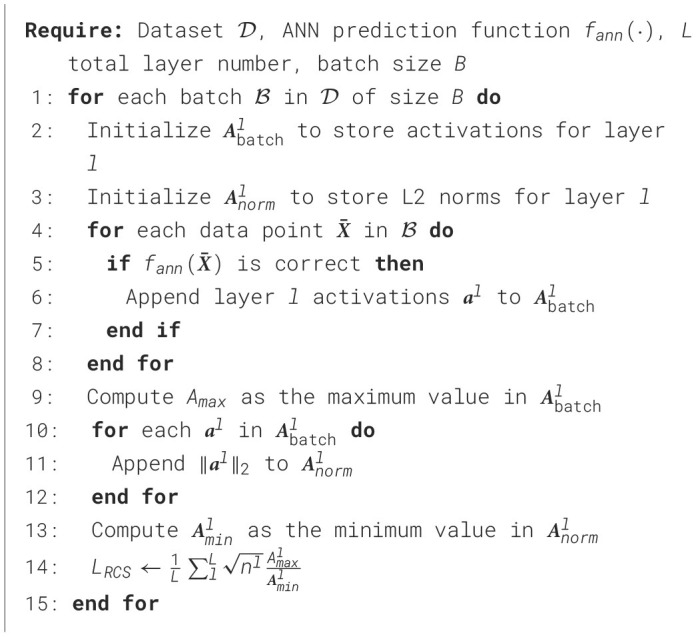
Compute RCS loss in ANN training.

#### 4.2.2 Regularizing direct training

In the case of direct training, SNN indicates its potential at processing spatiotemporal data, leading to a dynamic spiking rate $r_{d}^{l}\left(t\right)$ throughout the inference process. This capability, highlighted in Yao et al. (2021), sets direct training apart from ANN-to-SNN conversion, which is designed only for static input. Given that spikes can vary across timesteps in direct training, and their average may not accurately capture the temporal information, we approximate the spiking rate at each timestep by normalizing the membrane potential at that specific timestep. Thus, $r_{d}^{l}\left(t\right)$ is defined as


(15) $$\begin{array}{l} r_{d}^{l}\left(t\right)=\theta^{l}\left(t\right)+\frac{V^{l+}\left(t\right)}{V_{\text{thr}}^{l}}, \end{array}$$

where ***θ***^*l*^(*t*) is generated spikes and the normalized residual membrane potential $V^{l+}\left(t\right)/V_{thr}$ reflects the firing intention of neurons.

**Figure 4:**
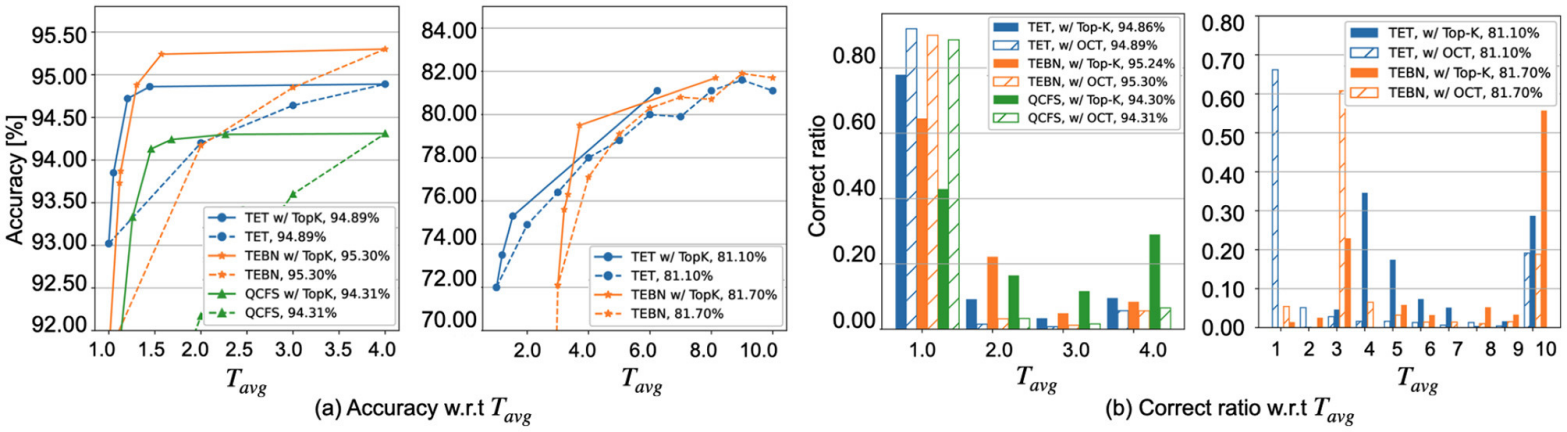
Comparison of SNN with and without Top-K cutoff on CIFAR10 (left) and CIFAR10-DVS (right) across various training methods: **(A)** The Top-K cutoff is determined by ϵ values ranging from 0.00 to 0.50 in increments of 0.05. **(B)** The statistical data is extracted from testing samples under ϵ = 0.02 for CIFAR10 ϵ = 0.0 for CIFAR10-DVS. The *x*-axis label has been revised.

In the next step, we focus on computing the desired spiking rate ${\tilde{r}}_{d}$. The trend where SNN accuracy improves more timesteps, which is attributed to the accumulation of relevant information over time, is illustrated in Figure 4 in Section 5. This observation forms the basis for our estimation of ${\tilde{r}}_{d}$, which is calculated as


(16) $$\begin{array}{l} {\tilde{r}}_{d}^{l}=\frac{1}{N}\overset{T}{\underset{t=T-N}{\sum }}r_{d}^{l}\left(t\right), \end{array}$$

where *N* is an integer hyperparameter. The proposed method posits that the later spiking rates can provide more representative information for a given sample. As indicated in Equation 6 from Section 4.1.1, consistent and correct predictions are crucial for optimal cutoff performance. Thus, we only optimize ***r***_*d*_(*t*) using Equation 10 only when the last *N* predictions are correct. The details of this approach are described in Algorithm 2.

**Algorithm 2:**
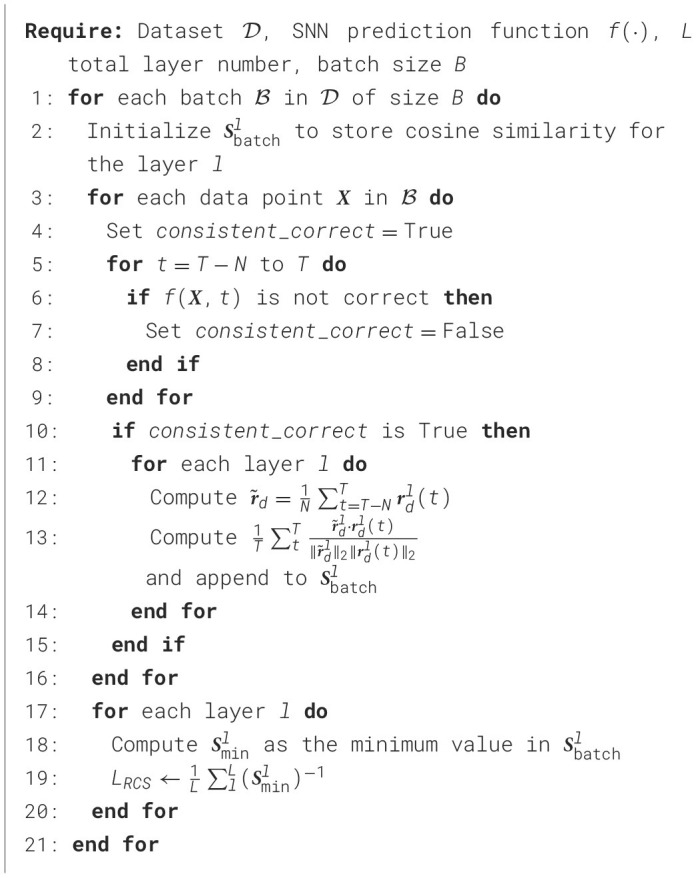
Compute RCS loss in SNN training.

#### 4.2.3 Optimization objective

Building on the *L*_*RCS*_ computed in Algorithms 1, 2 for ANN and SNN, respectively, we integrate the regularization term into our overall optimization objective for each model. *L*_*RCS*_ captures the worst-case features across layers during training, and minimizing *L*_*RCS*_ helps mitigate these issues. We followed Deng et al. (2022) and Bu et al. (2022), and we used cross-entropy loss (denoted as *L*_*ce*_) as the primary training loss. The final training objective with the RCS regularization is


(17) $$\begin{array}{l} \underset{W}{\min}\left(L_{ce}+\alpha L_{RCS}\right), \end{array}$$

where ***W*** is the weight matrix of the ANN or SNN to be trained, and α is a hyperparameter to the balance two loss terms.

## 5 Experiment

In this section, we discuss comprehensive experiments conducted to evaluate SNN models using our newly proposed metric “OCT,” in conjunction with the “Top-K” cutoff and the “RCS” regularization technique. These experiments were designed to explore the compatibility of the proposed approaches with prevalent SNN training methods. For example, in conversion-based training, we implement the quantised clip-floor-shift (QCFS) method proposed by Bu et al. (2022). The proposed method replaces ReLU with the QCFS activation function to reduce the loss of accuracy after conversion. For direct training, we adopted the temporal efficient training (TET) (Deng et al., 2022) and temporal efficient batch normalization (TEBN) (Duan et al., 2022), both of which represent the most recent developments in SNN training algorithms. For clarity, each configuration in our experimental setup is denoted within brackets: QCFS(·) indicates the quantization length, while TET(·) and TEBN(·) refer to the maximum timestep for training. Additionally, Top-K(·) indicates the setting of ϵ. For easy reference to the techniques incorporated in the models, we use notations such as “TET(·) + RCS, w/ Top-K,” indicating that the SNN model has been enhanced with both RCS regularization and the Top-K cutoff strategy. In our experiments, the ANN-to-SNN conversion method, such as QCFS, was applied only to frame-based datasets. We do not evaluate it on event-based datasets, which inherently involve temporal dynamicsbecause they focus primarily on spatial information.

### 5.1 Experimental datasets and setup

The experiments were conducted on various datasets, including both frame-based and event-based inputs, and network architectures. Specifically, we evaluated our approaches across diverse settings: ResNet-18 (He et al., 2016) for CIFAR10/100 (Krizhevsky et al., 2009) and Tiny-ImageNet (Le and Yang, 2015), VGGSNN (Deng et al., 2022) for CIFAR10-DVS (Li et al., 2017) and N-Caltech101 (Orchard et al., 2015), along with a five-layer convolutional network (Fang et al., 2021b) for DVS128 Gesture (Amir et al., 2017). To effectively process event-based datasets, we implement a downsampling strategy by integrating a 4 × 4 kernel with a stride of 4 at the beginning of the original network architecture. This adjustment can directly feed event data into the SNN, as suggested by Shrestha and Orchard (2018).

The samples in the event-based datasets record event addresses with on/off events over a specific period. The CIFAR10-DVS consists of 10,000 samples extracted from CIFAR10 (Li et al., 2017). Each sample has 128 × 128 spatial resolution. The length of each spike train is less than or equal to 1.3*s*. The N-Caltech101, dataset contains 8,709 samples categorized into 101 classes. The number of samples in each class ranged from 31 to 800. The length of each spike train was approximately 0.3*s*. The width in the *x*-direction does not exceed 240 pixels, and that in the *y*-direction does not exceed 180 pixels. For these two datasets, we used 90% samples in each class for training and 10% for testing. DVS128 Gesture comprises of 1,341 samples with 11 categories. Each sample is was analyzed for 6.0*s*. Due to repetitive information in these samples, the first 1.3 s were selected. All event-based samples were split into 10 frames for training and evaluation.

For training, the hyper-parameter α varied depending on the training method and dataset, and it was chosen from 0.001, 0.002, 0.003 for conversion-based methods and 0.001, 0.003, 0.005 for event-based datasets. For the frame-based datasets CIFAR10/100 and Tiny-ImageNet, we used a batch sizes of 128 and 300 epochs. The auto augmentation method proposed by Cubuk et al. (2019) was deployed on Cifar-10/100 to enhance the accuracy. For the event-based datasets, the training parameters were set to 100 epochs with batch sizes of 128 for CIFAR10-DVS, 64 for N-Caltech101, and 32 for DVS128 Gesture.

The evaluation of the Top-K cutoff requires a set of β values derived from the training dataset. To evaluate the efficiency, each sample is simulated until its corresponding OCT $\hat{t}$. We used *T*_avg_ to represent the average number of timesteps required for the inputs from the entire test dataset. As models often exhibit overconfidence post-training, we integrated dropout layers with a 0.3 dropout rate after each spiking layer to counteract this effect during characterization. The efficacy of this dropout is explained in Srivastava et al. (2014) and Jin et al. (2022). To implement RCS in direct training, Equation 16 suggests that the later spiking rates are expected to align with the desired spiking rates. Thus, we set *N* = 1 for *T* = 4 and *N* = 3 for *T* = 10, guided by the ratio *N*/*T*≈0.3.

### 5.2 Experimental results

Figure 4A demonstrates the implementation of the Top-K cutoff across various SNN training methods, demonstrating its ability to enhance computational efficiency by reducing the number of timesteps required to achieve comparable accuracy. Specifically, the SNN method requires **1.76 to 2.76 ×** **fewer timesteps** for CIFAR-10 with a near-zero accuracy drop of 0.01% to 0.06%, and **1.23 to 1.60 ×** **fewer timesteps** for CIFAR10-DVS with the same accuracy. However, while Top-K serves as a practical approximation, a discernible gap exists between this empirical approach and the theoretical cutoff by OCT. As illustrated in Figure 4, in Top-K cutoff effectively implements an adaptive timestep strategy but falls short of achieving the ideal cutoff, particularly in terms of correctly classifying samples at the first timestep compared to OCT. To provide further insight, we revisit Figure 3B from Section 4.1.2, which visually illustrates the impact of ϵ on Top-K cutoff across different samples. A larger ϵ may indicate fewer timestep for each sample; however, the accuracy is reduced. This highlights the need to optimize the Top-K cutoff during training via RCS.

Given that RCS is designed to complement the OCT, our experimental results consider the OCT as a key metric associated with Top-K on frame-based inputs (Table 1) and event-based inputs (Table 2). As seen in both tables, a smaller OCT always indicates better accuracy performance and a more effective cutoff.

**Table 1:** Comparison of accuracy and latency before and after applying RCS across different training methods on frame-based datasets.

**Dataset**	**Method**	**OCT**		**Top-K(0)**		**Top-K(0.05)**	
		**Acc**.	*T*_*avg*_	**Acc**.	*T*_*avg*_	**Acc**.	*T*_*avg*_
CIFAR10	QCFS(4)	94.04	1.27	94.04	4.00	94.04	2.40
	QCFS(4) + **RCS**	**94.32**	**1.26**	94.32	4.00	94.32	2.94
	TET(4)	94.89	1.20	94.89	4.00	94.86	1.45
	TET(4) + **RCS**	**95.24**	**1.19**	95.24	2.10	95.23	1.67
	TEBN(4)	95.30	1.22	95.30	4.00	95.24	1.57
	TEBN(4) + **RCS**	**95.49**	**1.22**	95.49	4.00	95.47	1.58
CIFAR100	QCFS(4)	75.20	2.03	75.20	4.00	75.20	3.80
	QCFS(4) + **RCS**	**76.21**	**2.00**	76.21	4.00	76.21	3.72
	TET(4)	77.02	1.84	77.02	4.00	77.02	3.30
	TET(4) + **RCS**	**77.81**	**1.81**	77.81	4.00	77.81	3.23
	TEBN(4)	77.93	1.85	77.93	4.00	77.93	3.03
	TEBN(4) + **RCS**	**78.13**	**1.85**	78.13	4.00	78.13	3.08
Tiny-ImageNet	QCFS(4)	47.11	2.97	47.11	3.98	47.11	3.91
	QCFS(4) + **RCS**	**47.71**	**2.94**	47.71	3.99	47.71	3.97
	TET(4)	56.56	2.52	56.56	3.74	56.56	3.65
	TET(4) + **RCS**	**56.69**	2.52	56.69	3.75	56.69	3.56
	TEBN(4)	56.19	2.56	56.19	3.87	56.19	3.63
	TEBN(4) + **RCS**	**56.69**	**2.54**	56.69	3.82	56.69	3.51

OCT reflects the theoretical cutoff performance, indicating undiminished accuracy and the average optimal cutoff timesteps. Top-K cutoff with ϵ values of 0.0 and 0.05 demonstrates the performance of the approximated cutoff. The bold values highlight the best results for each objective.

**Table 2:** Comparison of accuracy and latency before and after applying RCS across direct training methods on event-based datasets.

**Dataset**	**Method**	**OCT**		**Top-K(0)**		**Top-K(0.05)**	
		**Acc**.	*T*_*avg*_	**Acc**.	*T*_*avg*_	**Acc**.	*T*_*avg*_
CIFAR10-DVS	TET(10)	81.10	3.16	81.10	6.24	75.30	1.53
	TET(10) + **RCS**	**83.10**	**3.05**	83.10	6.10	76.90	1.43
	TEBN(10)	81.70	4.46	81.70	8.14	79.95	3.71
	TEBN(10) + **RCS**	**82.20**	**3.94**	82.20	7.88	79.10	3.27
N-Caltech101	TET(10)	85.01	2.64	85.01	7.39	83.48	1.96
	TET(10) + **RCS**	**85.66**	**2.57**	85.67	6.06	84.03	1.77
	TEBN(10)	82.49	3.01	82.49	7.44	79.32	1.86
	TEBN(10) + **RCS**	**83.15**	**2.99**	83.15	5.95	80.20	1.91
DVS128 Gesture	TET(10)	96.97	2.18	96.97	6.31	96.21	4.28
	TET(10) + **RCS**	**97.35**	**1.66**	97.35	5.14	95.83	2.53
	TEBN(10)	96.21	3.00	96.21	9.30	96.21	7.42
	TEBN(10) + **RCS**	**96.97**	**2.73**	96.97	9.10	96.97	7.21

The bold values highlight the best results for each objective.

In Table 1, RCS facilitates a significant OCT reduction for the QCFS models, ranging from 0.01 to 0.03 across CIFAR10/100 and Tiny-ImageNet. Specifically, on CIFAR100, this improvement was marked by a 0.3-fold decrease in OCT and a 1.01% increase in accuracy, thereby underlining the effectiveness of RCS in enhancing SNN from ANN training. Conversely, when RCS is applied to direct training methods, such as TET and TEBN, improvements in on frame-based datasets appear to be more modest. This limited improvement may be due to direct training methods are designed to optimize the network's performance at small timesteps, such as configuring a small maximum timestep (*T* = 4) during training. Furthermore, OCT estimates the theoretically optimal cutoff point (without accuracy loss), reflecting the upper bound of accuracy achievable during cutoff. In practical, the Top-K cutoff, as an approximation of OCT, achieves accuracy less than or equal to that of OCT, depending on the setting of ϵ.

**Figure 5:**
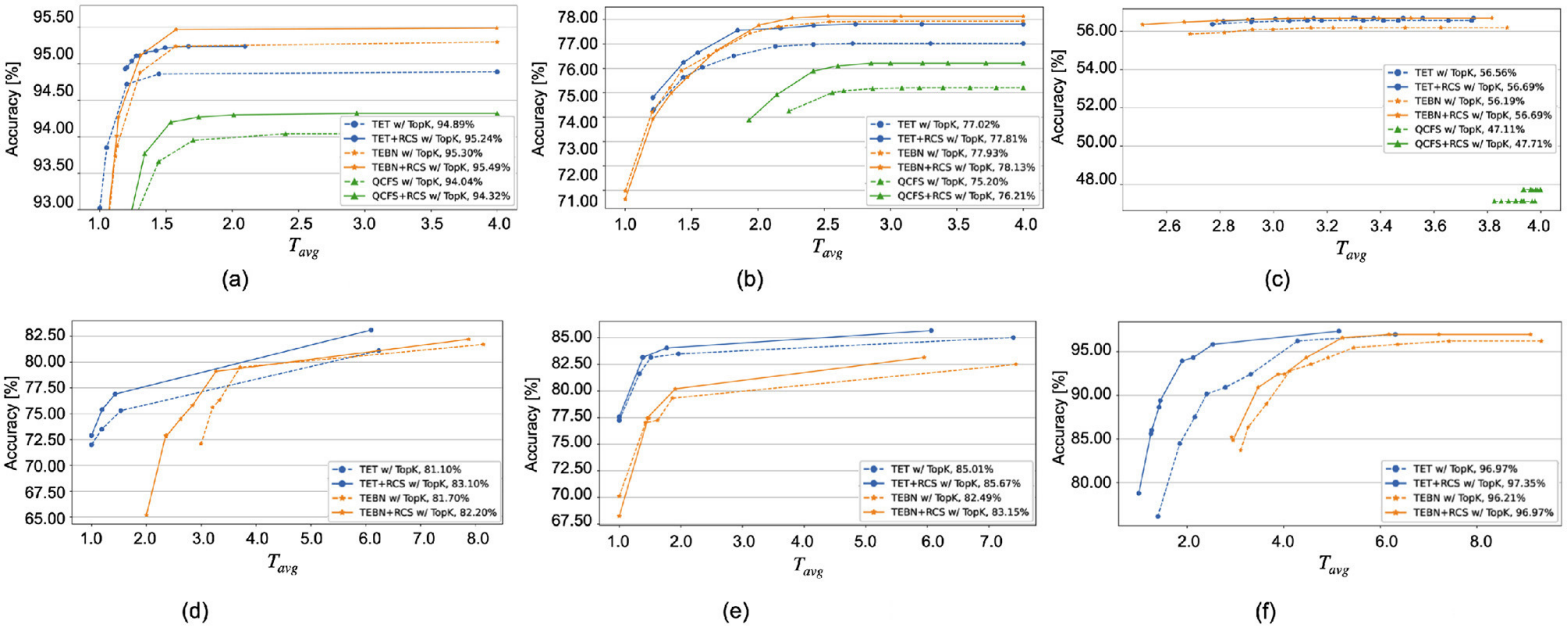
Comparison of Top-K cutoff accuracy before (dashed lines) and after (solid lines) regularization across a range of ϵ values from 0.00 to 0.50, increasing in steps of 0.05. The accuracy of the full-length input is detailed in the legend. The *x*-axis label has been revised. **(A)** CIFAR10. **(B)** CIFAR100. **(C)** Tiny-ImageNet. **(D)** CIFAR10-DVS. **(E)** N-Caltech101. **(F)** DVS128 Gesture.

The effect of RCS becomes more significant for event-based datasets, as shown in Table 2, with OCT reductions ranging from 0.02 to 0.52. For the TET with RCS, Top-K(0) enabled the SNN to achieve zero accuracy loss across all event-based datasets while requiring **1.64 to 1.95 × fewer timesteps**. This performance surpasses that of TET without RCS, which requires only **1.35 to 1.60 ×** **fewer timesteps**. Moreover, Figure 5 illustrates that the implementation of RCS shifts the accuracy curve upward compared to the curve without RCS, which means that the similar accuracy can have less inference timesteps.

### 5.3 Comparison with existing work on cutoff

Previous studies have investigated adaptive inference strategies for SNNs to improve inference efficiency. For instance, SEENN (Li et al., 2023b) employs reinforcement learning to train an auxiliary network jointly with the SNN, enabling early exits, whereas the dynamic confidence (DC) strategy (Li et al., 2023a) relies on post-training calibration.

**Figure 6:**
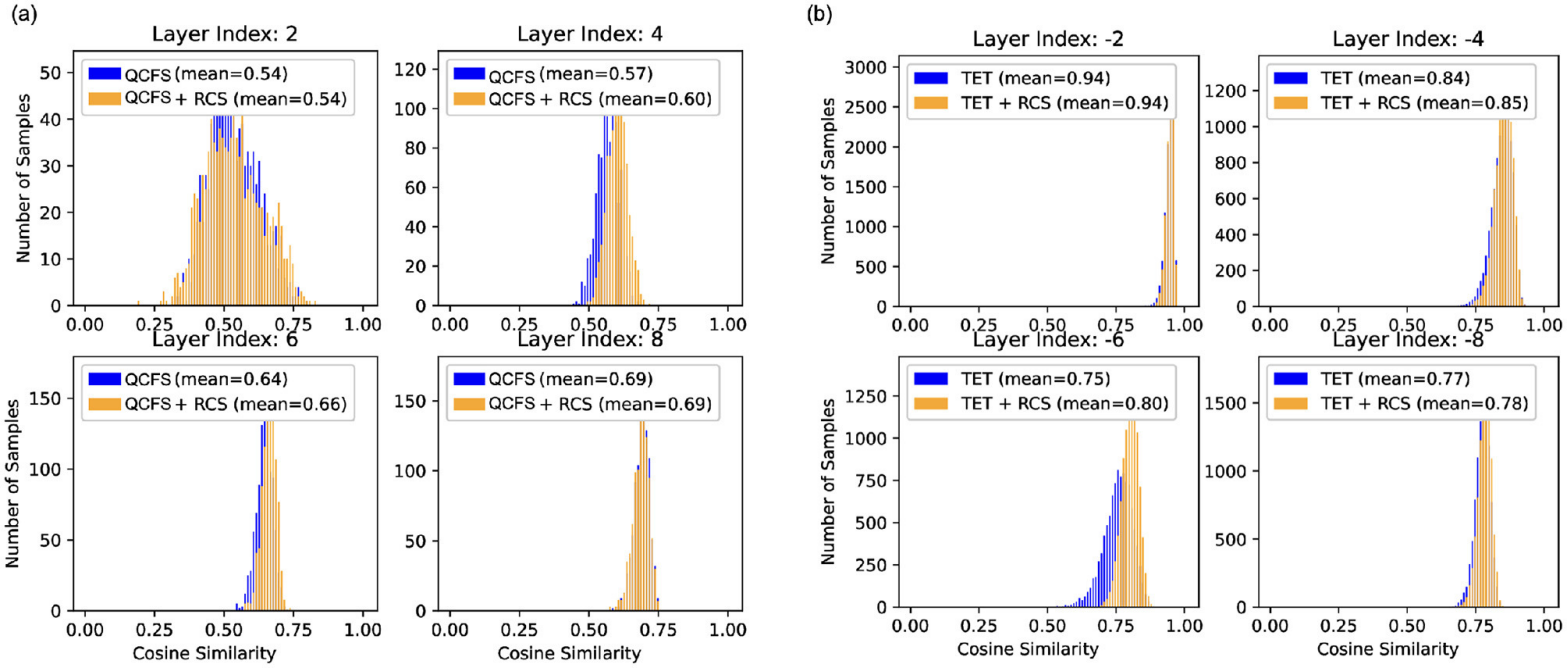
Histograms of cosine similarity at different layers for SNNs trained with and without the RCS regularizer. **(A)** Results for SNN (ResNet-18) trained with QCFS on CIFAR10 at *t* = 2. **(B)** Results for SNN (VGGSNN) trained with TET on CIFAR10-DVS at *t* = 3. Each subplot corresponds to a specific layer index. Note that layer index −2 indicates the second last layer.

In contrast, the proposed approach eliminates the need for auxiliary networks, offers a more efficient cutoff solution, and leverages RCS to assist training, enhancing cutoff robustness. Figure 6 shows the impact of RCS on feature alignment. The histograms show that RCS consistently improves cosine similarity across layers, indicating better alignment between the early timestep features and the expected features. Table 3 compares our results with those of SNN models that employ adaptive inference strategies. The results indicate that integrating the RCS and Top-K SNN cutoff techniques leads to superior performance at low timestep.

**Table 3:** Comparison of accuracy and latency between our methods and state-of-the-art SNN work.

**Dataset** **(Architecture)**	**Training** **Framework**	**Method**	**Acc. (*T*_*avg*, 1_)**	**Acc. (*T*_*avg*, 2_)**	**Acc. (*T*_*avg*, 3_)**
CIFAR10 (ResNet-18)	CFFS (Li et al., 2023a)	DC	94.11 (2.52)	–	–
	QCFS	DC	94.27 (11.51)	–	–
		SEENN-I	95.08 (2.01)	93.63 (1.40)	91.08 (1.18)
		RCS+Top-K	94.31 (1.71)	93.96 (1.46)	91.59 (1.12)
	TET	**RCS+Top-K**	**95.23 (1.58)**	**95.16 (1.32)**	**94.38 (1.07)**
CIFAR100 (ResNet-18)	QCFS	SEENN-I	65.48 (6.19)	56.99 (4.41)	39.33 (2.57)
		RCS+Top-K	76.21 (3.33)	75.22 (2.23)	71.94 (1.75)
	TET	RCS+Top-K	**77.81 (2.73)**	**77.57 (1.91)**	**76.17 (1.45)**
CIFAR10-DVS (VGGSNN)	TET	SEENN-I	82.7 (5.17)	77.60 (2.53)	–
		SEENN-II	82.6 (4.49)	–	–
		RCS+Top-K	**82.80 (4.14)**	**81.10 (2.52)**	**76.90 (1.43)**

The ϵ value for each model is adjusted within the range [0, 0.5] with step size of 0.01 to evaluate accuracy at specific timesteps. The bold values highlight the best results for each objective.

## 6 Conclusions

In this paper, we focus on developing an SNN that achieves high efficiency throughout both training and inference processes, making them particularly well-suited for inferring with adaptive timestep. We introduce two key innovations designed to enhance SNN performance: a regularizer targets the training phase, and a cutoff mechanism optimizes the inference stage. Our comprehensive experiments demonstrate these advancements, indicating notable enhancements in accuracy and inference efficiency over traditional methods. The Top-K cutoff technique introduced here proves to be versatile across various SNN neuron models, such as IF and LIF, provided the predictions rely solely on output analysis.

While our approach shows strong potential, some limitations remain. The current evaluation was primarily conducted on the VGG and ResNet architectures, and its applicability to advanced architectures like spiking transformers, has not been explored. Additionally, the regularizer increases memory requirements, as seen with a 26% increase in memory usage—from 8.9 to 11.3 GB—when directly training an SNN (*T* = 4) with ResNet-18 on CIFAR10. This overhead could pose challenges in training larger-scale networks. Furthermore, our evaluation relies on GPU-based simulations rather than hardware implementations.

Future studies could explore integrating these techniques with advanced architectures, such as spiking transformers. Expanding the evaluation to more complex datasets and real-world applications will provide a deeper understanding of the scalability and practical utility of the proposed methods. Further, optimizing the RCS to reduce its memory overhead would improve its suitability for larger-scale models, thereby addressing resource constraints. Finally, implementing and testing the cutoff with the SNN on Field Programmable Gate Arrays (FPGAs) provide valuable insights into their performance and feasibility for real-world applications.

## Data Availability

The original contributions presented in the study are included in the article/Supplementary material, further inquiries can be directed to the corresponding author.
