# Supplementary material for: Optimizing event-driven spiking neural network with regularization and cutoff
Source: Front Neurosci. 2025 Feb 19;19:1522788. doi: 10.3389/fnins.2025.1522788 (PMC11880274; doi:10.3389/fnins.2025.1522788)
Supplement: Supplementary file 1 [file Data_Sheet_1.pdf]

Optimising Event-Driven Spiking Neural Network with Regularisation and Cutoff: Supplementary Material

1 INEQUATION PROOF

We follow Banner et al. (2018) to derive the bound of expected norm of a random variable vector. By Jensen's inequality, it gives

$$\begin{aligned}\mathbb{E}(\|\boldsymbol{V}(t)\|_2) = \mathbb{E}(\sqrt{\sum_i V_i(t)^2}) &\leq \sqrt{\mathbb{E}(\sum_i V_i(t)^2)} \\ &= \sqrt{\sum_i \mathbb{E}(V_i(t)^2)}\end{aligned} \tag{S1}$$

As the $V_i(t)$ is a uniform random variable in range $[0, V_{\text{thr}}]$, the expected value of $V_i^2(t)$ can be computed as follows

$$\mathbb{E}(V_i(t)^2) = \int_0^{V_{\text{thr}}} x^2 \frac{1}{V_{\text{thr}}} dx = \frac{V_{\text{thr}}^2}{3} \tag{S2}$$

which yields

$$\mathbb{E}(\|\boldsymbol{V}(t)\|_2) \leq \frac{\sqrt{n}V_{\text{thr}}}{\sqrt{3}} \tag{S3}$$

Since t is constant value, the following inequality holds

$$\mathbb{E}(\|\boldsymbol{V}(t)/t\|_2) \leq \frac{\sqrt{n}V_{\text{thr}}}{\sqrt{3}t} \tag{S4}$$

2 SUMMARY OF NOTATIONS.

REFERENCES

Banner, R., Hubara, I., Hoffer, E., and Soudry, D. (2018). Scalable methods for 8-bit training of neural networks. *Advances in Neural Information Processing Systems* 31

Symbol	Definition
l	Layer index
L	Total number of layers
i	Element index
t	Discrete timestep
T	Maximum timestep
T_{avg}	The average number of timesteps
$\boldsymbol{W}$	Weight matrix
$\boldsymbol{V}^-$	Membrane potential before reset
$\boldsymbol{V}^+$	Membrane potential after reset
$\boldsymbol{Z}$	Weighted current
$\boldsymbol{\theta}$	Step function
V_{thr}	Threshold voltage
$\boldsymbol{b}$	Bias current
$\boldsymbol{X}$	Event input
$\bar{\boldsymbol{X}}_f$	Constant input current
$\boldsymbol{Z}_e^1$	Output of the 1st layer for event-based input
$\boldsymbol{Z}_f^1$	Output of the 1st layer for frame-based input
$\text{OCT}(\cdot)$	Optimal Cutoff Timestep (OCT) function
$\hat{t}$	Conditional discrete timestep
$f(\cdot)$	SNN prediction function
$\text{Top}_k(\cdot)$	Top-k function
$\boldsymbol{Y}$	Vector of output digits
D	Set of inputs
Y_{gap}	Gap between top-1 and top-2 output values
β	Threshold to trigger cutoff
$D\{Y_{\text{gap}} > \beta_t\}$	Set of inputs satisfying $Y_{\text{gap}} > \beta$
$C(t, D\{Y_{\text{gap}} > \beta_t\})$	Confidence rate at t with $Y_{\text{gap}} > \beta$ condition
ϵ	Pre-specified constant
$\boldsymbol{r}$	Average spiking rate
$\tilde{\boldsymbol{r}}$	Desired spike rate
$\lVert \cdot \rVert_2$	L2 norm
n	number of elements of a vector
$\boldsymbol{a}$	ReLU activation in ANN
$\boldsymbol{r}_c$	Spiking rate (ANN-to-SNN conversion)
$\tilde{\boldsymbol{r}}_c$	Desired spiking rate (ANN-to-SNN conversion)
$\boldsymbol{\Delta}$	Residual spiking rate (ANN-to-SNN conversion)
$\boldsymbol{r}_d$	Spiking rate (direct training)
$\tilde{\boldsymbol{r}}_d$	Desired spiking rate (direct training)
N	Integer hyperparameter to estimate $\tilde{\boldsymbol{r}}_d$
L_{CE}	Cross-entropy loss
L_{RCS}	Regulariser of Cosine Similarity loss
α	Hyperparameter to balance loss terms
